# Phytocontact Dermatitis due to Mustard Seed Mimicking Burn Injury: Report of a Case

**DOI:** 10.1155/2012/519215

**Published:** 2012-05-20

**Authors:** Hakan Yabanoglu, Sami Akbulut, Feza Karakayali

**Affiliations:** ^1^Department of Surgery, Faculty of Medicine, Baskent University, 06490 Ankara, Turkey; ^2^Department of Surgery, Diyarbakir Education and Research Hospital, 21400 Diyarbakir, Turkey

## Abstract

Mustard seeds have been used in traditional folk medicine as a stimulant, diuretic, and purgative and to treat a variety of ailments including peritonitis and neuralgia. Mustards are still used today in mustard plasters to treat rheumatism, arthritis, chest congestion, aching back, and sore muscles. To make a mustard plaster, mix equal parts of flour and powdered mustard and spread it as a paste on a doubled piece of soft cloth. Apply mustard plaster to the affected area for a maximum of 15 minutes. Prolonged application can result in burns to the skin and nerve damage. Skin lesions occur within hours after exposure, and there is no significant therapy procedure. This case report is about a patient with second-degree burn, occurred when a mixture including mustard seed was exposed to her skin in the pain therapy of the osteoarthritis in her left knee. There are no studies analyzing treatment of skin burns induced by mustard seed in the literature. While in this type of burns our experience is limited, we think that conservative approach should be first choice of treatment.

## 1. Introduction

Burn injuries can be encountered in all ages. The most common burn injuries among the Turkish population are caused by a variety of causes: fires, scalding substances, electricity, and chemical agents. When taking into account the mechanisms of chemical burns, it was observed that 4% of cases was caused by the application of herbs used as traditional medication. Despite the advances in medicine, a tendency towards using alternative treatments can be seen in every population, including the Turkish one, and plant application is among the most common methods used in folk medicine [1].

Sulphur mustard (SM) is a potential life-threatening chemical first was used during the first world war. SM has toxic effects on the skin, the respiratory system, and the eyes. SM is a chemically alkalizating agent that causes severe injuries when exposed to skin. SM's basic molecular target is the DNA, SM causes cross-reaction between the purine bases by alkalizing the DNA. This alkalization leads to the activation of poly ADP-ribose polymerase (parp) and this activation reduces the oxide nicotine amide dinucleotide phosphate formation in the cell. The loss of nicotine amide dinucleotide phosphate results in ATP-induced alterations in the keratinocyte functions and in the microflament structures and cause burn [2–4].

Mustard seeds are the small round seeds of various mustard plants. The seeds are usually about 1 or 2 mm in diameter. Mustard seeds may be colored from yellowish white to black. The seeds can come from three different plants: black mustard, brown Indian mustard, and white mustard. Mustards have been used in traditional folk medicine as a stimulant, diuretic, and purgative and to treat a variety of ailments including peritonitis and neuralgia. Mustards are still used today in mustard plasters to treat rheumatism, arthritis, chest congestion, aching back, and sore muscles. To make a mustard plaster, mix equal parts of flour and powdered mustard and spread it as a paste on a doubled piece of soft cloth. Apply mustard plaster to the affected area for a maximum of 15 minutes. Prolonged application can result in burns to the skin and nerve damage. Skin lesions occur within hours after exposure, and there is no significant therapy procedure. This case report is about a patient with second-degree burn occurred when a mixture including mustard seed was exposed to her skin in the pain therapy of the osteoarthritis in her knee.

## 2. Case Report

**Figure 1:**
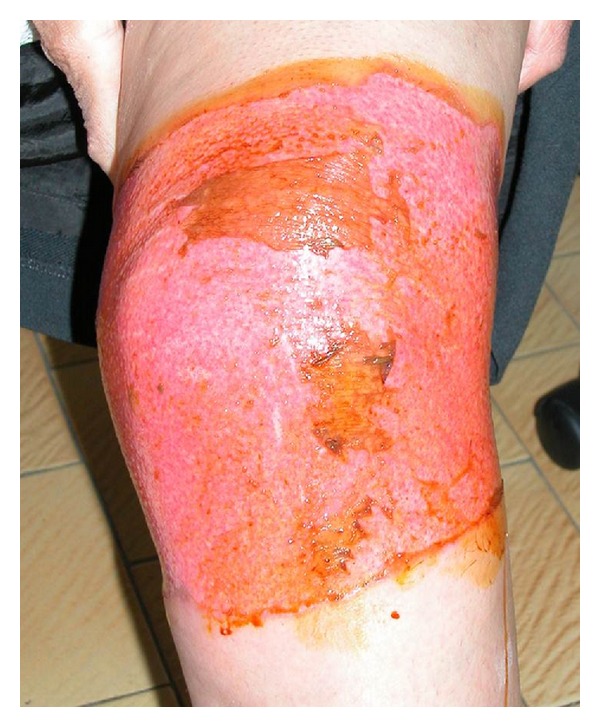
Second-degree skin burn due to mustard seed usage.

**Figure 2:**
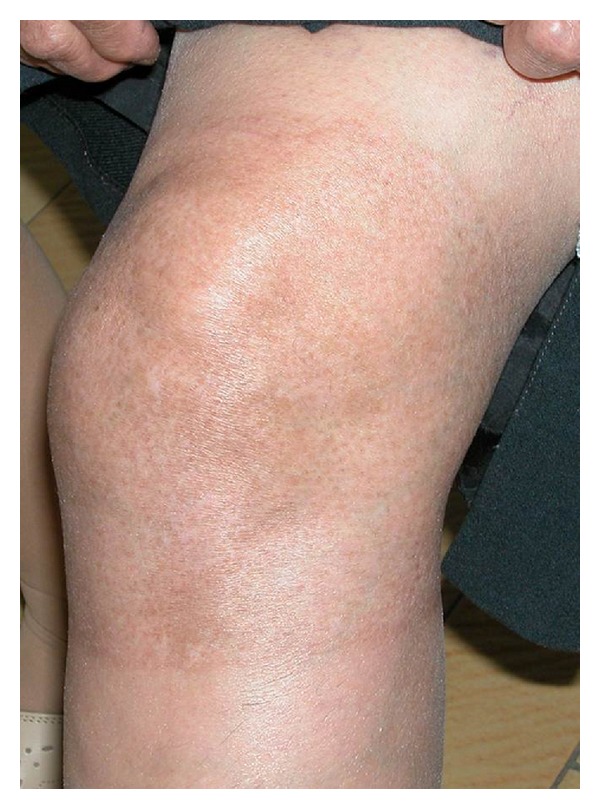
After conservative treatment.

A 71-years-old female patient was presented to our burn clinic with the complaints of pain, rush, and burning in the left knee. Except of hypertension regulated with amlodipine besylate, the patient had not any systemic disease such as diabetes mellitus or asthma. The patient stated that she was on medical therapy for a long time due to osteoarthritis, but she had stopped it both because of epigastric burning and pains on her left knee not to resolve. The patient had referred to a person dealing with alternative medicine on the advice from her neighbour. That person had applied vaseline to the left knee and then scattered mustard seeds that were pulverized in a mortar on the creamed area followed by covering it with cotton and gauze. The patient stated that she had opened the dressing after 24 hours because of she felt burn in the left knee and that she had washed her knee which was slightly rushed using water and soap. However, complaints of rush, swelling, and itching in the left knee had started at the 36 hour after the application. On her physical examination, an erosion was found, surrounding the knee and compatible with second-degree burn, being more in the anterior side (Figure 1). The treatment was planned as the patient to be hospitalized in the burn unit since she was elderly and the burn was surrounding the joint. First the burn area was cleansed with distilled water and debridement was made through a scalpel. Following application of the silver sulfadiazine cream, the burn area was wrapped with a gauze. This treatment was planned as once a day and the patient was discharged after one week. Topical silver sulfadiazine cream and daily dressing changes were applied over 14 days. The burn area was completely healed at the end of the first month (Figure 2). No contracture developed during the 3-month follow-up period.

## 3. Discussion

SM is a chemical weapon that was used during the first world war by the Germans against the French troops. SM is a chemical agent that causes skin, lung, and eye injuries when exposed with its liquid and high vapour concentrations. It also has carcinogenic and mutagenic effects. It is an alkalizating agent and its exposure to human skin may cause severe injuries. It makes the epidermis separated from the dermis at the dermal epidermal junction [3, 4]. Skin lesions usually occur within hours after exposure; eritema and the swellings occur first and then followed by the ulcer and the necrosis [2]. Energy loss is the responsible reason for this cell necrosis. Because of the dividing basal cells, skin becomes an important target for the SM. SM affects the epithelial tissues around the exposure site by its high chemical reactivity. Skin burns, caused by chemicals such as SM, may diffuse to nearby tissues and cause biochemical alterations at these sites. Despite the effects on the skin, the eyes, the pulmonary and gastrointestinal systems, and the most important complication is the bone marrow depression [2].

Skin burn appears within hours after exposure and improves rapidly. Burns with SM have longer hospitalization periods when compared with other thermal burns. Ampiric and symptomatic therapies are the most common management procedures. Recent studies proved that new techniques such as mechanic dermabrasion and laser debridement, that improve injury healings by removing the injured tissues, are useful, and animal model studies showed that these techniques have an improving effects on injury healings [5]. In another case/control study comparing the dermabrasion and the control groups, it is found that reepithelization time was faster in the dermabrasion group. According to our report, dermabrasion is an effective method in surgical treatment of SM burns [6].

There are no studies analyzing treatment of skin burns induced by SM in the literature. In our study medical therapy reached success in a short time. While in this type of burns our experience is limited, we think that conservative approach should be first choice of treatment.
